# Magnetic resonance imaging brain atrophy assessment in primary age-related tauopathy (PART)

**DOI:** 10.1186/s40478-019-0842-z

**Published:** 2019-12-09

**Authors:** Miguel Quintas-Neves, Merilee A. Teylan, Lilah Besser, João Soares-Fernandes, Charles N. Mock, Walter A. Kukull, John F. Crary, Tiago Gil Oliveira

**Affiliations:** 1 Division of Neuroradiology, Hospital de Braga, Braga, Portugal; 2 Department of Epidemiology, National Alzheimer’s Coordinating Center, University of Washington, Seattle, Washington USA; 3 School of Urban and Regional Planning, Institute for Human Health and Disease Intervention, Florida Atlantic University, Boca Raton, Florida USA; 4 Department of Pathology, Nash Family Department of Neuroscience, Friedman Brain Institute, Ronald M. Loeb Center for Alzheimer’s Disease, Icahn School of Medicine at Mount Sinai, New York, NY USA; 5 Life and Health Sciences Research Institute (ICVS), School of Medicine, University of Minho, Campus Gualtar, 4710-057 Braga, Portugal; 6 ICVS/3B’s—PT Government Associate Laboratory, Braga/Guimarães, Portugal

**Keywords:** Primary age-related tauopathy, Alzheimer disease, MRI, Brain imaging

## Abstract

Alzheimer disease (AD) is a neurodegenerative disorder characterized pathologically by the accumulation of amyloid-beta (Aβ) plaques and tau neurofibrillary tangles (NFTs). Recently, primary age-related tauopathy (PART) has been described as a new anatomopathological disorder where NFTs are the main feature in the absence of neuritic plaques. However, since PART has mainly been studied in post-mortem patient brains, not much is known about the clinical or neuroimaging characteristics of PART. Here, we studied the clinical brain imaging characteristics of PART focusing on neuroanatomical vulnerability by applying a previously validated multiregion visual atrophy scale. We analysed 26 cases with confirmed PART with paired clinical magnetic resonance imaging (MRI) acquisitions. In this selected cohort we found that upon correcting for the effect of age, there is increased atrophy in the medial temporal region with increasing Braak staging (*r* = 0.3937, *p* = 0.0466). Upon controlling for Braak staging effect, predominantly two regions, anterior temporal (*r* = 0.3638, *p* = 0.0677) and medial temporal (*r* = 0.3836, *p* = 0.053), show a trend for increased atrophy with increasing age. Moreover, anterior temporal lobe atrophy was associated with decreased semantic memory/language (*r* = − 0.5823, *p* = 0.0056; and *r* = − 0.6371, *p* = 0.0019, respectively), as was medial temporal lobe atrophy (*r* = − 0.4445, *p* = 0.0435). Overall, these findings support that PART is associated with medial temporal lobe atrophy and predominantly affects semantic memory/language. These findings highlight that other factors associated with aging and beyond NFTs could be involved in PART pathophysiology.

## Introduction

Degenerative dementias share the common feature of protein misfolding in the brain, resulting in a defining dementia-type abnormal protein with typical topographic deposition. One such example is the accumulation of neurofibrillary tangles (NFTs), composed of hyperphosphorylated tau protein, on a subgroup of neurodegenerative diseases termed tauopathies [14]. Alzheimer disease (AD) is the most studied tauopathy; in this particular case, another hallmark is the presence of extracellular amyloid-beta (Aβ) plaques and brain atrophy of specific regions, such as the medial temporal lobe [14]. Following several brain autopsy studies [28, 29] from which AD-indistinguishable NFTs were found in the absence of Aβ plaques, the term primary age-related tauopathy (PART) was proposed for this group of patients [10].

The pathological diagnosis of PART requires the presence of tau positive NFT with a Braak stage ≤ IV, with minimal deposition beyond medial temporal lobes, and with sparse (“possible PART”) to absent (“definite PART”) Aβ deposition [10]. Concomitantly, PART can be clinically classified as “asymptomatic” (without cognitive deterioration or dementia) or “symptomatic” (with cognitive deterioration or dementia) [16]. Since PART is essentially a histological designation, there is a need for further studies to advance in vivo diagnostic tools to effectively differentiate it from AD, which could also lead to better selection of patients for clinical trials. Therefore, neuropsychological evaluation, cerebrospinal fluid biomarkers assessment and brain magnetic resonance imaging (MRI) are important tools to consider in the characterization of PART.

To clinically differentiate PART from AD, previous studies assessed the relationship between clinical and neuropathologic features. It was observed that Braak staging was associated with an increased odds of having cognitive impairment [7], but comparing to AD, patients with PART were shown to have relative sparing of semantic memory/language [9]. Moreover, it was shown that episodic memory is relatively spared in patients with the moderately severe stratum with a Clinical Dementia Rating (CDR®) of 0.5–1, and attention is relatively spared in the most severe stratum with CDR of 2–3 [9]. Consistent findings were described in a clinical-imaging-pathological study that showed that aggregated tau distribution was associated with poorer cognitive performance in “definite” PART patients [20]. Moreover, a recent study showed that autopsy confirmed PART cases with mild cognitive impairment or dementia were clinically diagnosed with AD greater than 50% of the time [30]. Therefore, there is a clinical necessity of ante-mortem criteria for the effective diagnosis of PART, for instance, using brain MRI. To date, only one study compared the in vivo brain MRI to neuropathological findings in patients with “definite” PART, which found an association between Braak staging and atrophy of the left head of the hippocampus [20]. Also, it described an antero-posterior gradient of the hippocampus, with relative sparing of the posterior portion, a finding that significantly correlated with the preservation of episodic memories on neuropsychological tests [20]. However, no systematic imaging interpretation method to efficiently use in a clinical setting was proposed.

Therefore, the main goal of our study was to compare in vivo brain MRI findings with neuropathological findings at time of autopsy for people with “definite” PART pathology. Specifically we used previously validated visual atrophy scales that evaluate six different brain regions [13] to assess the potential link of NFT with typical brain imaging pathological patterns, controlling for either Braak staging or age. Moreover, we inquired which neuropsychological domains were specifically correlated with atrophy in the affected brain regions.

## Materials and methods

### Participants

Cross-sectional data from the National Alzheimer’s Coordinating Center’s (NACC) Uniform Data Set (UDS) and Neuropathology Data Set (NP) were used. Data contributed to NACC, including demographic, neuropsychological, clinical, and diagnostic data on participants with normal cognition, mild cognitive impairment, and dementia, were collected since 2005 from approximately 30 past and present Alzheimer's Disease Centers (ADC) in the United States. The UDS and NP data have been meticulously described [4, 5, 8, 23]. We used data collected between September 2005 and March 2018. All participants provided written informed consent at each ADC.

### Selection criteria

From the total number of UDS participants as of the March 2018 data freeze (*n* = 37,568), only those with neuropathological data (*n* = 5135), most specifically neuritic plaque (NP) data (*n* = 5110) were enrolled. We excluded participants: (a) with neuropathological evidence of frontotemporal lobar degeneration, Lewy bodies, amyotrophic lateral sclerosis, prion disease, or argyrophilic grains; (b) with clinical evidence of dementia with Lewy bodies, Parkinson disease, Down syndrome, Huntington disease, prion disease, corticobasal degeneration, or progressive supranuclear palsy. With the application of these exclusion criteria, 2350 participants remained. Considering only those with “definite” PART (defined as no NP – *n* = 394) and availability of MRI scans, 33 participants were considered. Five patients with other lesions that biased volumetric interpretation (e.g., brain tumors) were excluded. Two patients less than 40 years old were excluded, in order to obtain a more homogeneous cohort in terms of age. Therefore, our cohort was composed of 26 patients.

### Neuropathology data

Neuropathological data was collected by the ADCs by using a standardized Neuropathology Form on UDS patients who died and consented to autopsy and neuropathologic examination. Details on brain tissue preparation and staining within the NACC NP dataset have been previously described [8]. This data was used to establish the participants with “definite” PART, and to obtain the Braak stage for neurofibrillary degeneration.

### Brain MRI data

MR imaging examinations were performed mostly on 1.5 T or 3 T scanners, both from Philips®, Siemens® or GE® manufacturers. Several imaging protocols were used by the different centres; we always used T1-weighted sequences in order to establish the degree of brain atrophy – spin-echo (SE) or magnetization-prepared rapid gradient-echo (MPRAGE) sequences.

### Image analysis

We used a group of validated MRI visual rating scales [13] in order to assess brain atrophy on the following regions: anterior cingulate, orbito-frontal, anterior temporal, fronto-insular, medial temporal, and posterior regions. Briefly, as previously described by the simplified version of these scales: orbito-frontal and anterior cingulate regions were rated on the first anterior slice where the corpus callosum becomes visible, both from “0–3”, where “0” is representing no atrophy; the fronto-insular was rated over three slices, starting on the first anterior slice where the anterior cingulate becomes visible and moving posteriorly, from “0–3”; the anterior temporal was rated at the level of the temporal pole, just anterior to where the “temporal stem” connects the frontal and temporal lobes, from “0–4”; the medial temporal was rated according to the medial temporal lobe atrophy (MTA) score – performed on the hippocampus at the level of the anterior pons, from “0–4”; the posterior region was rated according to the four-point posterior atrophy scale described by Koedam, from “0–3” [21] – overall score based on the presence of atrophy in sagittal (widening of the posterior cingulate and parieto-occipital sulcus, and atrophy of the precuneus on left and right by considering paramedian sagittal images), axial (widening of the posterior cingulate sulcus and sulcal dilatation in parietal lobes on axial images) and coronal (widening of the posterior cingulate sulcus and parietal lobes on coronal images) orientations, assessed for left and right separately [13]. Two independent classifiers with neuroradiology experience were responsible for rating the images. For each brain region scale, an average of both hemispheres was considered and an average of both classifiers was used. In this specific cohort no significant asymmetric atrophy was observed (Additional file 1: Figure S1). In order to aid the rating process, reference images for each rating scale were provided to the classifiers based on Harper et al. [13]. Atrophy classification data is summarized in Additional file 1: Table S1. For Fig. 4, scale values were then converted to percentage values, from 0%, as no atrophy, to 100%, as maximal atrophy value considered for each scale. Images were visualized in Horos® imaging software version 3.0.1.

### Neuropsychological data

Several tests of memory, executive function, language and processing speed were considered in our analysis. Executive function was assessed by the Trail Making Test (TMT) A and B, which globally tests attention, visual scanning and search skills, and psychomotor speed and coordination [26]; TMT A can independently assess processing speed, while TMT B assesses set switching; on both parts of this test (i.e., A and B), the total number of seconds to complete the test, the number of commission errors, and number of correct lines were recorded; the Wechsler Adult Intelligence Scale digit symbol test was also considered to provide an estimate of processing speed [31]. Semantic memory/language was assessed by category (vegetables and animals) verbal fluency [22], consisting of a test on registering the total number of vegetables and animals named in 60 s; the Boston naming test [11], which also assesses the effect of language function, more precisely the confrontational word retrieval, was included in this evaluation, and consisted of showing pictures (up to 60) to the patient, and wait up to 20 s for the patients to name them. Attention and working memory was evaluated by Digit span forwards and backwards test [18], consisting on registering the ability of recalling a sequence of numbers shown, and the total length of numbers successfully achieved. Episodic memory was assessed by Current Logical Memory 1A Story Units Recalled [1], for the total number of items recalled. Mini-Mental State Examination [12] was performed as a brief cognitive screening instrument that provides a measure of global cognition. Concerning the data used for this study, the average delay between MRI and neuropsychological data acquisition was 2.7 years. All valid and available neuropsychological data retrieved from NACC concerning the PART population under study was included for analysis and is summarized in Additional files 1: Table S2.

### Statistical analysis

In order to assess the rating acuity, Spearman’s correlation coefficient was calculated for the pair of classifiers per each region and it was confirmed that there was a statistically significant association between both (Additional file 1: Table S3). Residuals were calculated for brain regional atrophy scores controlling for either age or Braak staging. These were then plotted independently to Braak staging and age, respectively. Regional brain atrophy scores were also plotted for each of neuropsychological test considered and only significant associations were represented. Pearson correlation coefficients were calculated (*r*) and statistically significant values were considered for *p* < 0.05. SPSS® Statistics version 25, and GraphPad® Software version 8.0.0 were used for all analyses.

## Results

Twenty-six participants were considered for this study. Table 1 summarizes the demographic characteristics of the enrolled participants. Of the 26 “definite” PART patients, 17 (65.4%) were male. The mean age at the time of MRI examination was 80.6 years (range: 51–99), and the mean age of death was 83.5 years (range: 51–101). The mean Braak stage was 1.5 (range: 0–4).

**Table 1:** Demographic characteristics of definite PART patients

	PART population (*N* = 26)
Age MRI	80.6 (51–99)
Age of death	83.5 (51–101)
Difference age MRI to death	2.96 (1–7)
Braak stage	1.5 (0–4)
Sex	
Male	17 (65.4%)
Female	9 (34.6%)

The table shows the summarized demographic characteristics of a cohort of 26 anatomopathological confirmed PART cases. Age MRI is the subject age when brain imaging acquisition was performed and is reported in years as a continuous variable with average, minimum and maximum values represented. Age of death is reported in years as a continuous variable with mean, minimum and maximum values represented. Braak stage is represented as categorical values based on an anatomopathological validated staging scale with mean, minimum and maximum values represented. Sex is differentially represented as male and female with frequency and relative percent values for each

**Fig. 1:**
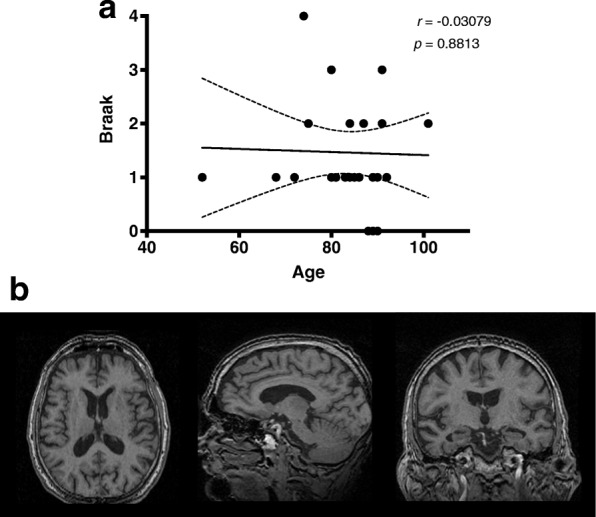
Distribution of cases from a definite PART cohort based on age and Braak staging. **a** Age in years is represented based on age of death of the subjects. Braak staging is represented as categorical values based on an anatomopathological validated staging scale. **b** Representative case of a 78 year-old male from the PART cohort. Axial, sagittal and coronal MRI T1-weighed images are shown

**Fig. 2:**
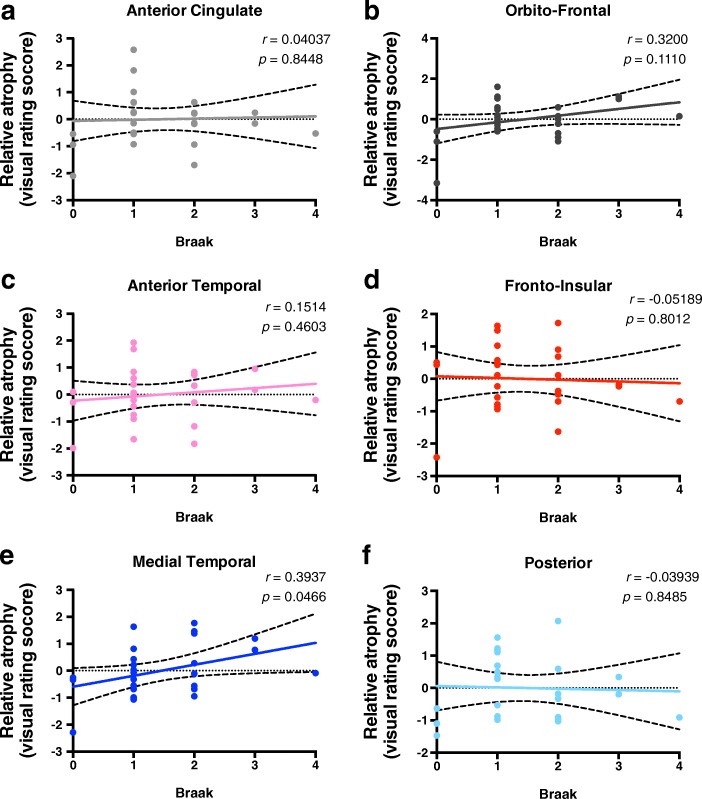
Medial temporal lobe atrophy correlates with increasing Braak staging. The values represented are residuals corrected for age, with Pearson correlation analysis between Braak staging and atrophy of different brain regions based on a previously validated imaging rating scale. The regions evaluated are **a** anterior cingulate, **b** orbito-frontal, **c** anterior temporal, **d** fronto-insular, **e** medial temporal and **f** posterior brain regions. Statistical significance was considered as *p* < 0.05

**Fig. 3:**
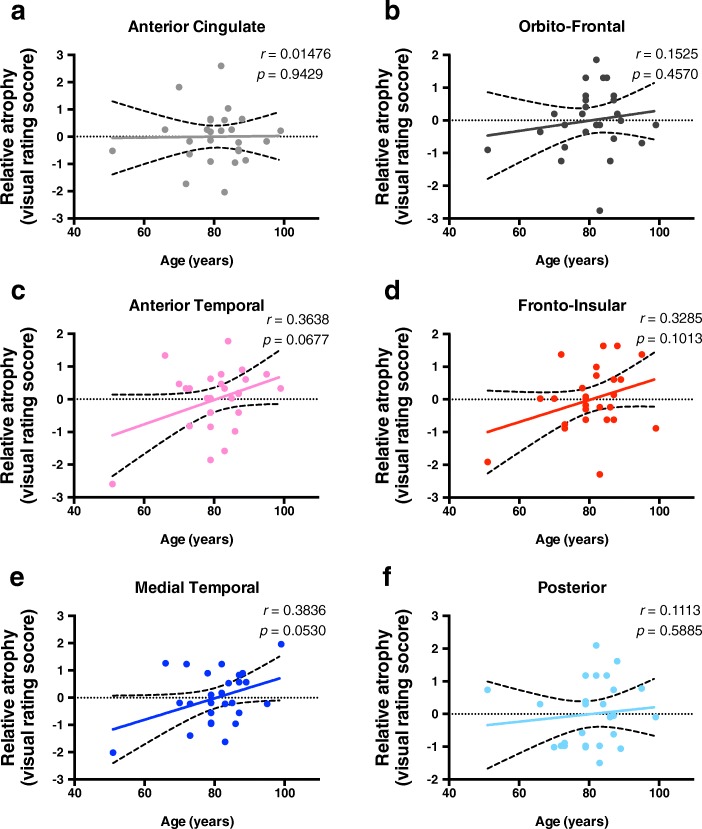
Anterior and Medial temporal lobe atrophy increases with increasing age. The values represented are residuals corrected for Braak staging, with Pearson correlation analysis between age and atrophy of different brain regions based on a previously validated imaging rating scale. The regions evaluated are **a** anterior cingulate, **b** orbito-frontal, **c** anterior temporal, **d** fronto-insular, **e** medial temporal and **f** posterior brain regions. Since statistical significance was considered as *p* < 0.05, no significant correlations were found, although both anterior temporal (*r* = 0.3638, *p* = 0.0677), and medial temporal (*r* = 0.3836, *p* = 0.053) regions were found to be close to this statistical threshold

In this specific cohort, even though there was no significant correlation between age and Braak stage (*r* = − 0.03079, *p* = 0.8813) (Fig. 1a) the majority of cases had some degree of regional atrophy (Fig. 1b), and no significant changes were observed between men and women (Additional files 1: Figure S2). Upon correction for age, the relative percentage of atrophy of the medial temporal lobe significantly correlated with Braak staging (*r* = 0.3937; *p* = 0.0466) (Fig. 2e). No statistically significant results of regional brain atrophy were found for the other regions evaluated (Fig. 2). Upon correction for Braak staging, we observed a trend to increasing atrophy with age, predominantly on the anterior temporal (*r* = 0.3638, *p* = 0.0677) (Fig. 3c) and medial temporal (*r* = 0.3836, *p* = 0.053) regions (Fig. 3e). Moreover, when we compared cases Braak ≤1 with Braak ≥2 we observed significant increased atrophy score in the medial temporal region (Additional file 1: Figure S3).

**Fig. 4:**
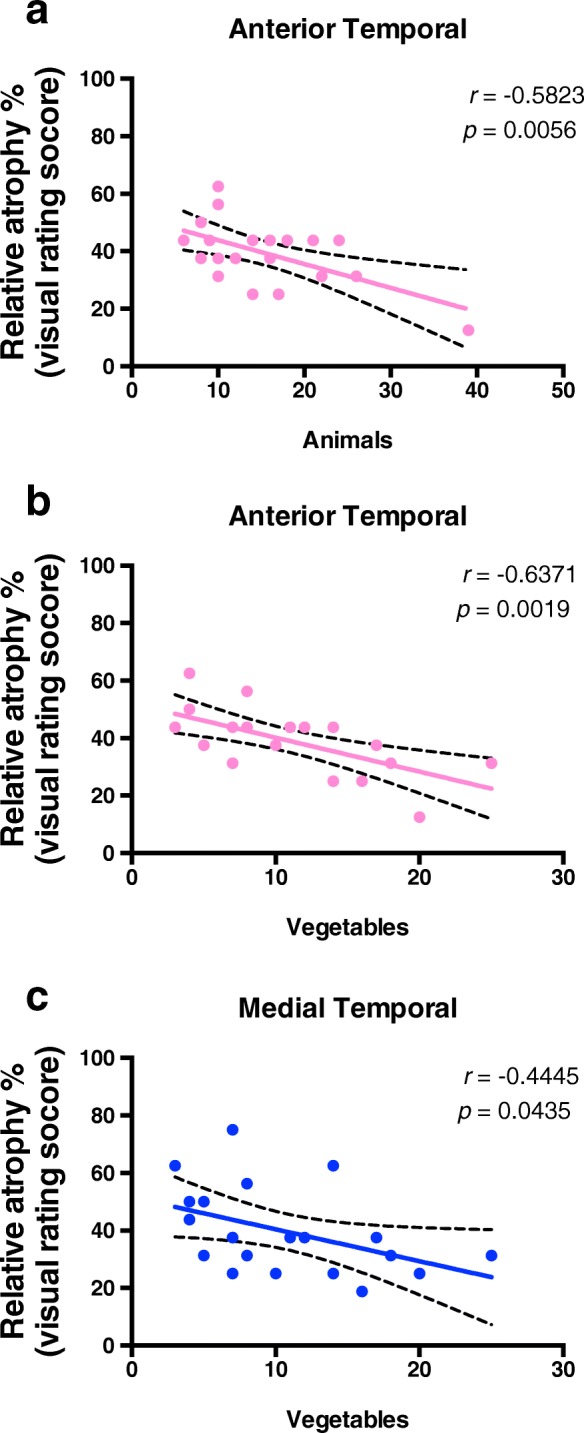
Semantic memory/language impairment correlates with anterior and medial temporal atrophy. Pearson correlation analysis of neuropsychological test performance versus temporal brain atrophy regions evaluated with a previously validated imaging rating scale for patients with definite PART was performed and only the statistically significant correlations are shown (*p* < 0.05). “Vegetables” represents the number of vegetables a subject can name in 1 min. “Animals” represents the number of animals a subject can name in 1 min. **a** Anterior temporal lobe atrophy is associated with decreased semantic memory/language, given by the animals (*r* = − 0.5823, *p* = 0.0056) and **b** vegetables naming tests (*r* = − 0.6371, *p* = 0.0019). **c** Medial temporal lobe atrophy is also associated with decreased semantic memory/language, given by the vegetables naming test (*r* = − 0.4445, *p* = 0.0435)

From the 26 cases, the majority of them also performed an extensive neuropsychological battery testing. Considering this sample, we checked for an association between regional brain atrophy and performance in each of the cognitive domains assessed. We observed that atrophy of the anterior temporal lobe was associated with decreased semantic memory/language, given by the animals and vegetables naming tests (*r* = − 0.5823, *p* = 0.0056; and *r* = − 0.6371, *p* = 0.0019, respectively) (Fig. 4a, b), as was atrophy of the medial temporal lobe, given by the vegetables naming test (*r* = − 0.4445, *p* = 0.0435) (Fig. 4c).

## Discussion

The main goal of this study was to compare the in vivo brain MRI findings to neuropathological findings at autopsy for patients identified with “definite” PART pathology. More specifically, we assessed the effects of Braak staging and age on PART brain imaging associated atrophy patterns and its potential implications for specific neuropsychological deficits. Our results showed an association between Braak staging and atrophy of the medial temporal lobe on “definite” PART patients (i.e., brains without neuritic plaques) upon correction for age. This main finding was consistent with one described by another imaging study, on which left hippocampal volume was found to be decreased in PART [20]. It is also consistent with previous anatomopathological studies describing PART, where NFTs accumulation is predominantly confined to the temporal lobe [10]. Importantly, medial temporal lobe atrophy is commonly found in typical AD, namely with decreased hippocampal volume [25]. While at the imaging level this observation does not help completely to distinguish PART from early stage AD, it might distinguish it from more advanced cases of AD where atrophy is extensive in other brain regions. Given the sparsity of PART patients with Braak stages III and IV, this is an assumption future studies with more patients on these advanced stages should address.

Our findings partly explain why many PART cases identified post-mortem were clinically diagnosed as AD [30]. The combination of other features, such as neuropsychological specific presentation, CSF signatures or PET could lead to a more fine-tuned distinction between PART and AD.

Various other observations support PART as a separate pathological entity: the absence of an association between PART and APOE ε4 allele, the strongest risk factor for AD [3, 15]; “ghost” tangles, i.e. extracellular tangles, are more frequently found in PART than AD patients [17]; and patients diagnosed with PART are frequently in the 8th–9th decades, as was found in our study, making it highly improbable that Aβ deposition would only start then [20]. In fact, our results support the hypothesis of PART being a form of pathological brain aging, with preferential atrophy in the medial temporal lobes.

After controlling for Braak staging, our observations indicate that, in this selected cohort, aging leads to a higher degree of atrophy with predominance over the anterior and medial temporal lobe regions – a fact that is consistent with previous reported effects of aging [27]. One hypothesis is that other neuropathological factors associated with aging and beyond NFTs could be contributing to this regional temporal atrophy, one possibility being transactive response DNA-binding protein 43 (TDP-43). Interestingly, a recently described neuropathologic entity, limbic-predominant age-related TDP-43 encephalopathy (LATE), which is characterized by TDP-43 accumulation in the limbic system, can also present with dementia symptoms and brain atrophy, with potential involvement of the temporal lobe [24]. Remarkably, not only was TDP-43 pathology associated with greater atrophy in the medial temporal lobe regions in PART cases [19], but also independently of other neuropathological conditions [6]. Therefore, future studies should address the independent or cumulative impact of Aβ, tau and TDP-43 on brain regional atrophy, using AD, PART and LATE cohorts. Even though major vascular lesions were excluded after clinical neuroradiologist assessment, it is still possible that finer microvascular lesions, which are partially identified as white matter T2 hyperintensities, could be another potential contributing factor addressed in future studies.

We further inquired as to which neuropsychological domains could be impaired with the observed atrophy in the temporal lobe. Indeed, anterior temporal lobe atrophy significantly correlated with worse performance on animals and vegetables naming, i.e., semantic memory/language domain tests, as also did medial temporal lobe atrophy with vegetables naming. These findings are consistent with a previous study that stated the importance of temporal lobe on semantic verbal fluency [2]. Moreover, our results are consistent with a previous study that showed predominant anterior hippocampus atrophy on patients with PART, which also presented semantic memory/language domain deficits [20]. Another key point is that, despite using a different brain atrophy assessment methodology (i.e. visual rating scales), our observations are somewhat consistent with previous reports using voxel-based morphometry [20]. Also, we believe our study provides evidence for the use of a clinical setting-applicable visual rating scale that can efficiently assess brain regional atrophy by a trained neuroradiologist. This could be paramount to routine clinical practice, given the limited time and resources to continuously post-process volumetric acquisitions. Since another study found that semantic memory/language was relatively preserved in PART compared to AD patients [9], future imaging studies should tackle the question of potential different patterns of atrophy in PART and AD, and its correlation with neuropsychological presentation.

While this study had some important strengths, such as the confirmed neuropathologic data and the usage of an easy to apply and interpret atrophy rating score, there are some important limitations. First, it was based on a small, convenience, autopsy-based sample, a factor that limits its generalizability to other populations. The NACC database, in general, has limitations of its generalizability, as the participants tend to be more white and more affluent than the population as a whole. However, there was no additional selection bias in choosing the participants for this study, as we chose all eligible participants (i.e. people who had neuropathologically defined PART and who had an MRI available). Second, despite using validated MRI rating scales on patients with dementia, qualitative assessments and rating are almost invariably associated with inter-observer variability. However, given that observers applying these ratings should have several years of neuroradiology experience, we believe that in the clinical setting this could be overcome. Third, despite always using T1-weighted images on MRI visual rating, not all were volumetric acquisitions, introducing a source of variability in this study. Fourth, the variability in scanner manufacturers and field strengths used on the acquisition of images is also a potential source of variability in the interpretation and rating of the brain regional atrophy. Finally, another limitation is that the subset of subjects that were included in this study had incomplete information on other co-morbidities that could be associated with brain atrophy, such as hypertension or diabetes mellitus, or on other neuropathologic features, such as TDP-43 pathology.

## Conclusion

In summary, our findings provide evidence that in “definite” PART patients, i.e. brains with tau deposition without neuritic plaques, there is a correlation between atrophy of the medial temporal lobe and Braak staging. Moreover, since we show that aging leads to temporal lobe atrophy, independently of Braak, other factors beyond tau tangles could be contributing to atrophy. Finally, we provide evidence of a potential deficit on semantic memory/language domain in these patients. Future studies with volumetric imaging acquisitions should focus on differences in brain regional atrophy between PART and AD patients.

## Supplementary information

**Additional file 1.** **Table S1.** Atrophy Classification in PART population. **Table S2.** Neuropsychological evaluation in PART population. **Table S3.** Inter-rater analysis. **Figure S1.** No asymmetric atrophy observed. Comparison between right and left hemisphere regional atrophy ratings was performed. The regions evaluated are **a** anterior cingulate, **b** orbito-frontal, **c** anterior temporal, **d** fronto-insular, **e** medial temporal and **f** posterior brain regions. **Figure S2.** No sex differences in atrophy. Comparison of regional atrophy ratings was performed between men and women cases. The regions evaluated are **a** anterior cingulate, **b** orbito-frontal, **c** anterior temporal, **d** fronto-insular, **e** medial temporal and **f** posterior brain regions. **Figure S3.** Higher Braak score shows higher atrophy in the Medial Temporal region. Comparison between cases with Braak ≤ 1 and Braak ≥ 2. The regions evaluated are **a** anterior cingulate, **b** orbito-frontal, **c** anterior temporal, **d** fronto-insular, **e** medial temporal and **f** posterior brain regions. * *p *< 0.05.

## Data Availability

The datasets used and/or analyzed during the current study are available upon request on the NACC database.
