# Supplementary material for: Magnetic resonance imaging brain atrophy assessment in primary age-related tauopathy (PART)
Source: Acta Neuropathol Commun. 2019 Dec 9;7:204. doi: 10.1186/s40478-019-0842-z (PMC6902469; doi:10.1186/s40478-019-0842-z)
Supplement: Supplementary file 1 — Additional file 1. Table S1. Atrophy Classification in PART population. Table S2. Neuropsychological evaluation in PART population. Table S3. Inter-rater analysis. Figure S1. No asymmetric atrophy observed. Comparison between right and left hemisphere regional atrophy ratings was performed. The regions evaluated are a anterior cingulate, b orbito-frontal, c anterior temporal, d fronto-insular, e medial temporal and f posterior brain regions. Figure S2. No sex differences in atrophy. Comparison of regional atrophy ratings was performed between men and women cases. The regions evaluated are a anterior cingulate, b orbito-frontal, c anterior temporal, d fronto-insular, e medial temporal and f posterior brain regions. Figure S3. Higher Braak score shows higher atrophy in the Medial Temporal region. Comparison between cases with Braak ≤ 1 and Braak ≥ 2. The regions evaluated are a anterior cingulate, b orbito-frontal, c anterior temporal, d fronto-insular, e medial temporal and f posterior brain regions. * p < 0.05. [file 40478_2019_842_MOESM1_ESM.pdf]

Supplementary Table 1. Atrophy Classification in PART population

	**PART population** (N=26)
**Anterior cingulate**	1.35 (±0.12)
**Orbito-frontal**	1.48 (±0.09)
**Anterior temporal**	1.52 (±0.11)
**Fronto-insular**	1.71 (±0.15)
**Medial temporal**	1.51 (±0.14)
**Posterior**	1.57 (±0.11)

Atrophy classification of different brain regions was performed using a previously validated scale system (Harper et al.). Mean values are reported for each region, with standard error of mean in parentheses.

Supplementary Table 2. Neuropsychological evaluation in PART population

	**PART population**
**MMSE** (N=21)	26.57 (±0.79)
**LOGIMEM** (N=20)	10.95 (±1.39)
**MEMUNITS** (N=20)	9.3 (±1.59)
**MEMTIME** (N=20)	21.35 (±1.19)
**DIGIF** (N=21)	7.71 (±0.49)
**DIGIFLEN** (N=21)	5.9 (±0.29)
**DIGIB** (N=21)	5.86 (±0.56)
**DIGIBLEN** (N=21)	4.1 (±0.34)
**ANIMALS** (N=21)	15.52 (±1.69)
**VEGETABLES** (N=21)	10.76 (±1.3)
**TRAILA** (N=22)	51.14 (±5.14)
**BOSTON** (N=21)	23.8 (±1.11)

Several tests of memory, executive function, language and processing speed were performed for a subset of PART subjects (N=20-22). Mean values are reported for each region, with standard error of mean in parentheses. MMSE: Mini–Mental State Examination, 30 – Total score; LOGIMEM: total number of story units recalled from this current test administration; MEMUNITS: Logical Memory IIA — Delayed — Total number of story units recalled; MEMTIME: Logical Memory IIA — Delayed — Time elapsed since Logical Memory IA — Immediate; DIGIF: Digit span forward trials correct; DIGIFLEN: Digit span forward length; DIGIB: Digit span backward trials correct; DIGIBLEN: Digit span backward length; ANIMALS: Total number of animals named in 60 seconds; VEGETABLES: Total number of vegetables named in 60 seconds; TRAILA: Trail Making Test Part A — Total number of seconds to complete; BOSTON: Boston Naming Test, 30 — Total score.

Supplementary Table 3. Inter-rater analysis

	**Rho**
**Anterior cingulate**	0.378**
**Orbito-frontal**	0.293*
**Anterior temporal**	0.584***
**Fronto-insular**	0.633***
**Medial temporal**	0.68***
**Posterior**	0.706***

Inter-rater analysis of both classifiers for atrophy classification of different brain regions using a previously validated scale system (Harper et al.). Spearman correlation analysis was performed and Rho values are shown. * $p < 0.05$, ** $p < 0.01$, *** $p < 0.001$.

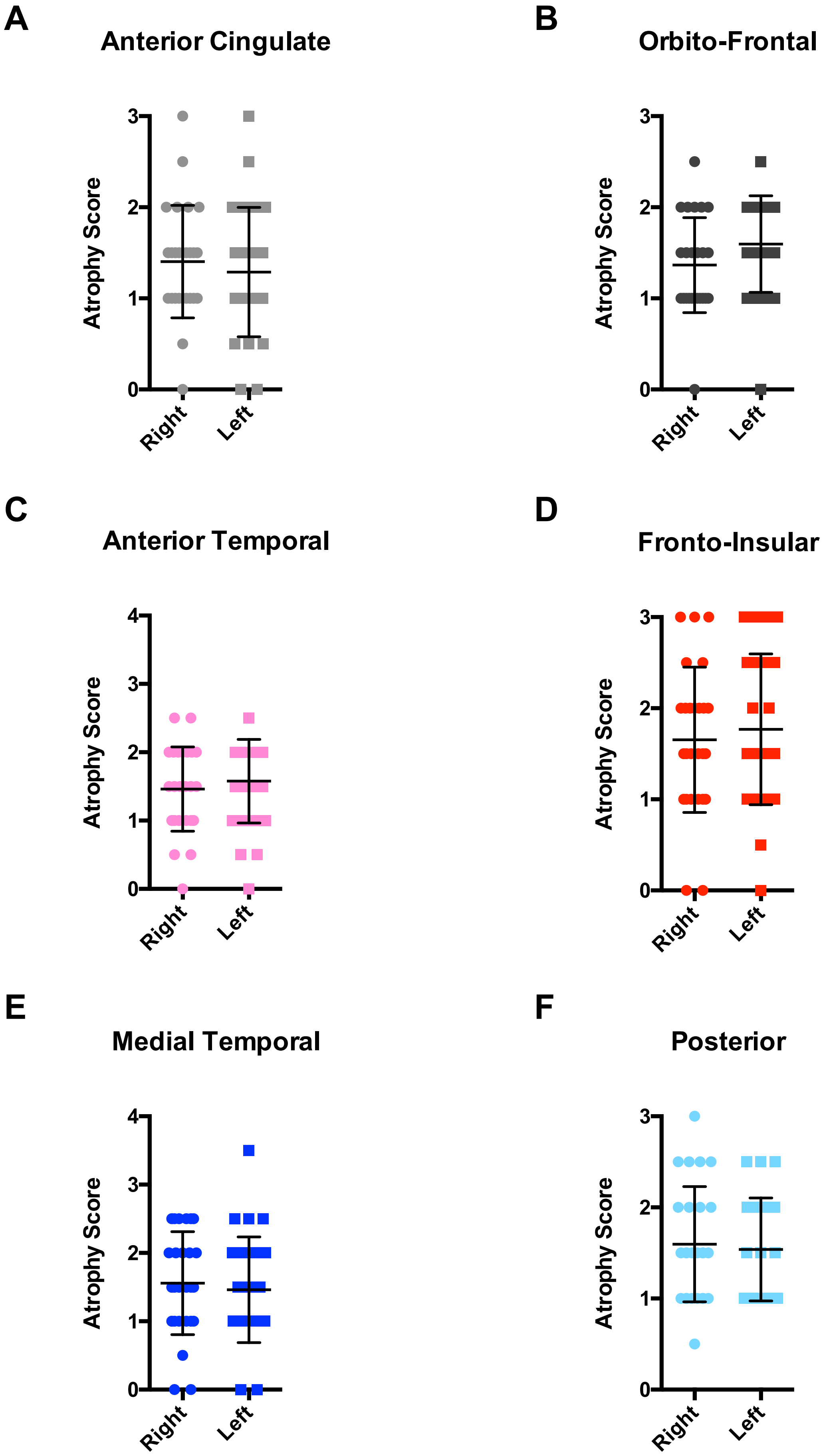

Supplementary Figure 1

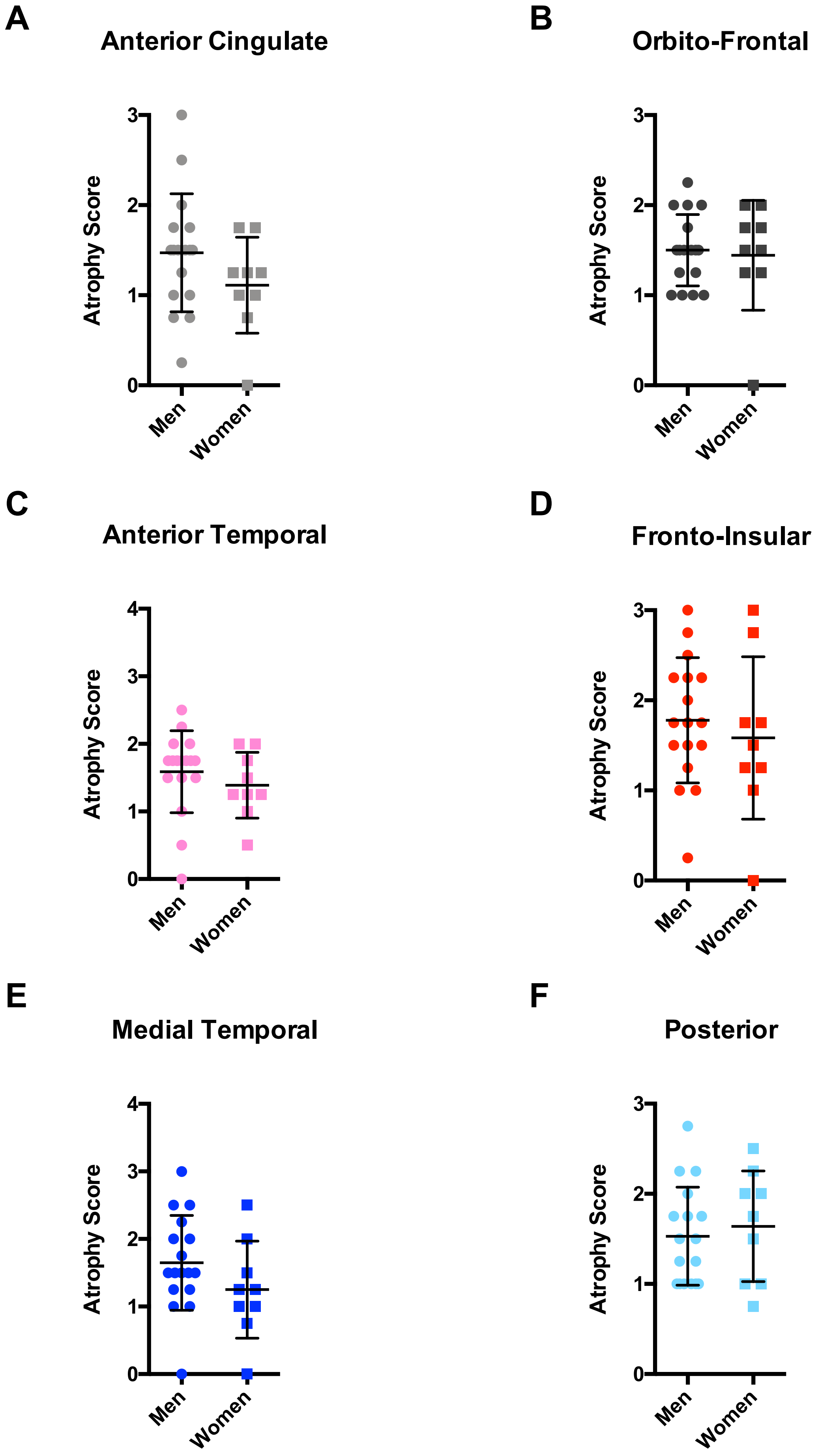

Supplementary Figure 2

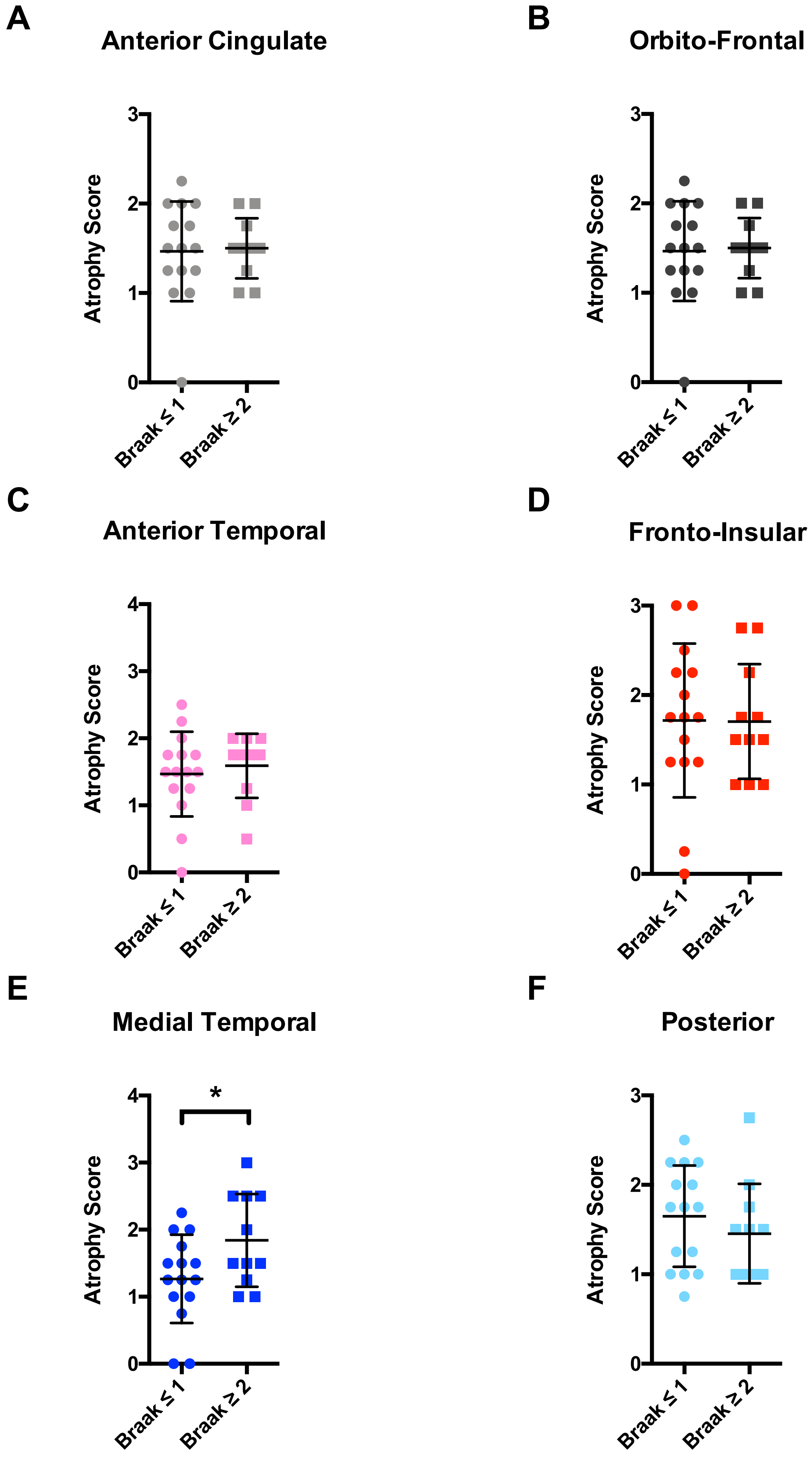

Supplementary Figure 3
